# Impact of Asthma Education Program 2020-2021 on Asthma Control Among Bronchial Asthma Children in Madinah City, Saudi Arabia

**DOI:** 10.7759/cureus.40571

**Published:** 2023-06-17

**Authors:** Jawharah Alhazmi, Shaima Alhazmi, Enas Alharbi, Areej Alghamdi, Rawan Alrumaithi, Mohammed Altamimi, Shahad Alharbi, Bader Aljohani, Faisal Alghamdi

**Affiliations:** 1 Pediatric Medicine, King Salman bin Abdulaziz Medical City, Madinah, SAU; 2 Pediatric Medicine, Ohud Hospital, Madinah, SAU; 3 Allergy and Immunology, King Salman bin Abdulaziz Medical City, Madinah, SAU; 4 Preventive Medicine, King Salman bin Abdulaziz Medical City, Madinah, SAU; 5 Anesthesia, Al-Madinah Al-Munawarah Hospital, Madinah, SAU; 6 Primary Health Care, Quba Primary Health Care Center, Madinah, SAU; 7 Clinical Nutrition, College of Applied Medical Sciences, Taibah University, Madinah, SAU; 8 Family Medicine, Madinah Health Cluster, Madinah, SAU; 9 Pediatrics, King Salman bin Abdulaziz Medical City, Madinah, SAU

**Keywords:** saudi arabia, education, asthma control, children, asthma

## Abstract

Background: Asthma control among asthmatic children still remains suboptimal. Saudi literature are scarce in this context, and there is a paucity of reports that compare asthma control level pre- and post-education program directed to asthmatic patients and their parents.

Objectives: The objective of this study was to assess the impact of asthma education and flu vaccination on asthma control in asthmatic children in Madinah region from 2020 to 2021, in terms of ED visits, hospitalization, pediatric intensive care unit (PICU) admission, and asthma control level.

Methods: A cross-sectional study was conducted at primary health care (PHC) centers in Al-Madinah City, Saudi Arabia. The study analyzed data from 804 asthmatic children patients from randomly selected six PHC centers. The data were collected by a valid structured questionnaire. The questionnaire included socio-demographic and clinical data. Child asthma symptoms control was examined by the Childhood Asthma Control Test (C-ACT) for children aged 5-12 years, and the Test for Respiratory and Asthma Control in Kids (TRACK) for children less than five years of age. The collected data were analyzed using the appropriate statistical tests.

Results: The mean age of the studied children was 6.1±3.0 (1-14 years), with 59.8% of them being males. There have been statistically significant reductions for asthmatic children in ED visits/month, hospitalization, and pediatric ICU admission/years in the post-education groups for all studied patients and patients <five years and five to 12 years. The average ACT and TRACK scores were significantly higher in the post-education group. The controlled asthma was also significantly higher among post-education groups. The rate of ED visits, hospitalization, critical care needs, and PICU admission significantly decreased among children receiving flu vaccination.

Conclusion: This study emphasizes the role of asthma education and flu vaccination in improving asthma control status of asthmatic children and in decreasing the rate of ED visits, hospitalization, and critical care needs. More longitudinal and experimental studies are needed to confirm these findings.

## Introduction

Asthma is a chronic respiratory disease that impacts the quality of life and affects 7-79% of population in different countries according to Global Initiative for Asthma (GINA) [1]. In Saudi Arabia, asthma is one of the most common chronic diseases, with an increasing prevalence during the past years. The overall prevalence of asthma among Saudi children showed high regional variation with a cumulative prevalence rate ranging from 9.5% to 13.4% [2]. In Madinah city, Saudi Arabia, a prevalence of 23.6% was reported, where asthma was more common in younger children [3].

Asthma has a significant impact on children, their families, and the community as a whole in terms of school absence days, poor quality of life, frequent emergency department (ED) visits, hospitalizations, and pediatric intensive care unit (PICU) admission, and deaths [4,5]. In a systematic review of the economic burden of asthma, hospitalization was found to cost up to 86% of all asthma-related costs, and poor asthma control was associated with an increased cost of care [6,7].

One of the major components of asthma management is patient education. Asthma education programs (AEPs) aim to help patients recover symptoms, follow treatment plans, control environmental triggers, and seek medical care when symptoms get worse [7,8].

Influenza vaccine also is a part of the asthma education program as national recommendations from the Centers for Disease Control and Prevention include the guidance that everyone aged six months and older should be vaccinated against the flu. Children younger than five years of age and children with asthma are at increased risk for serious flu complications, including inflammation of the airways, worsening asthma symptoms, or pneumonia [9]. It is well-documented in public health and health promotion field that structured health education programs given to the right participants in the right settings, improve health behavior, social support, and health internality [8].

Current guidelines recommend family empowerment through education at every opportunity across the healthcare continuum. The National Asthma Education and Prevention Program, led by inpatient asthma nurse practitioners (IANPs), combines several teaching strategies for parents, improving their skills and knowledge in controlling asthma [10,11]. The Saudi Initiative for Asthma (SINA) has also emphasized the role of patient and family knowledge on a good quality of life of school-age children with asthma [11]. Moreover, studies have suggested that when patients receive a good education and instructions on asthma, proper nutrition, and vaccination against viral infections including influenza, they can manage mild attacks at home, which results in a significant decrease in the number of absenteeism days and daytime asthma symptoms [10].

Most of the previous Saudi literature focused on establishing the prevalence of asthma in children and the reasons behind it. On the other hand, however, few studies have focussed on the impact of education and self-management of asthma among school children. From this point of view, this study aimed to assess the impact of asthma education programs and flu vaccination on asthma control children in Madinah region from 2020 to 2021, in terms of emergency department (ED) visits, hospitalization, pediatric intensive care unit (PICU) admission, and asthma control level.

## Materials and methods

A cross-sectional study with pre- and post-interventional design was carried out at primary health care (PHC) centers in Madinah region, Saudi Arabia, during the year 2020-2021, to assess the impact of asthma education programs on asthma control among Saudi children attending primary health care centers.

Madinah city is the second holy city and the fourth largest city in Saudi Arabia. It is located in the Hejaz region of western Saudi Arabia, and the capital of Al Madinah Province with a total population of 1.5 million [12]. Madinah is divided into four health sectors belonging to the Ministry of Health and includes 36 PHC centers.

Medical records of 1107 asthmatic patients aged 1-14 years were obtained, who are visiting the emergency department (ED) of one of the two hospitals Madinah Maternity Children Hospital (MMCH) or Ohud hospital, or visited the ED of the chosen six primary health care centers during the period from November 1, 2018, to the end of October 2020 were eligible to be included in the study. Newly diagnosed asthmatic children, children with co-morbidities, those with no history of hospitalization, ED visits, and those who refused to receive the education materials were excluded from the study.

After the informed consent was obtained, initially a trained physician interviewed the eligible parents to be assessed by completing the comprehensive asthma patient assessment form which included the clinical data (emergency department {ED} visits, hospital admission, and pediatric intensive care unit {PICU} admission, duration of illness and identifying trigger) [13], then filling the questionnaire for child asthma symptoms control by using the Childhood Asthma Control Test (C-ACT) for children aged five to 12 years, and Test for Respiratory and Asthma Control in Kids (TRACK) for children less than five years of age, all data collected as pre-education date as baseline [14].

The C-ACT is an Arabic version questionnaire that was developed and validated for use among children aged five to 12 years. The test consisted of seven questions - four questions for child response (parent's help in case of child needs help to read or understand) and three questions for parents. The control of symptoms and severity of asthma is made on a five-point scale ("not controlled at all," "poorly controlled," "somewhat controlled," "well controlled," and "completely controlled") and a total score ranging from zero to 25. The total Asthma Control Test (ACT) scores up to 5 means "not controlled" and 25 means "best controlled" with a cutoff point of 15 to differentiate between controlled and not controlled asthma. The Test for Respiratory and Asthma Control in Kids (TRACK) used for children under five years of age consists of five questions to be answered by the parent of asthmatic child, with scores ranging from 0 to 20, and a total scoring ranging from zero to 100, with a cutoff point of 80 to differentiate controlled from not controlled asthma.

After that children voluntarily received influenza vaccination, the educational materials according to the last updated evidence from Saudi Initiative for Asthma (SINA) and the Global Initiative for Asthma (GINA) guideline in the Arabic language were given to children patients and their parents at the pediatric clinics through a hard copy or QR code (including 5 minutes video motion graphic and 20 infographics about bronchial asthma definition, exacerbation management, home management of bronchial asthma {BA} exacerbation action plan, infographics about influenza vaccine, and general information about upper respiratory tract infection {URTI}), and home visits were allowed if needed. The studied patients who received the educational materials were followed up by phone in the second and third sessions of the study year to collect the post-education data about the outcome variables.

Data analyses were performed by using SPSS software version 22.0 for Windows (Chicago, IL: SPSS, Inc.). The baseline data were tabulated and presented for 1077 asthmatic children by using frequency number and percentage for categorical variables and mean and standard deviation for quantitative variables. The final analysis, however, was done on 804 asthmatic children. The excluded 273 children were distributed as follows: associated co-morbidities (n=45), not using inhaler (n=85), lost to follow-up (n=69), those with no history of emergency department (ED) visits and/or hospitalization (n=40), and those refused to receive education materials or missed follow-up (n=34). Dependent chi-square test and dependent t-test were used to compare the distribution of the studied outcome variables (emergency department {ED} visits/month, hospitalization/years, pediatric intensive care unit {PICU} admission/years, and average Asthma Control Test {ACT} and Test for Respiratory and Asthma Control in Kids {TRACK} scores among them before and after health education program). Comparison of asthma control and other outcome variables by the vaccination status of the studied children was done by using independent chi-square and independent t-tests. The level of statistical significance was defined as p≤0.05.

Ethical approval

Approval was taken from the local Ethics Committee, and work approval was officially taken from the deputy of the Health Directorate for Primary Health Care (PHC) in Madinah, Saudi Arabia. Ethical consideration was considered to ensure the confidentiality and privacy of the collected data.

## Results

The mean age of the studied children was 6.1±3.0 years and ranged from 1-14 years. The male children were representing 59.8% of the studied sample and the majority (93.7%) were of Saudi nationality. Cough and shortness of breath were representing 60.4% and 58.7% of asthma manifestation among them, respectively. Almost all studied patients were on short-acting beta agonists (SABA) medication with the relief reported in 78.9% of them. The use of systematic corticosteroids was found in 24.2%, associated co-morbidities in 4.1%, positive family history of asthma and other allergic diseases in 69.4%, and flu vaccination was reported by 47.8% of the studied children. The demographic and clinical characteristics of the studied asthma children showed in Table 1.

**Table 1:** Socio-demographic and clinical characteristics of the studied asthma children (n=1077). *Data are presented by n (%) and mean±SD. **The number and percent exceeded 1077 (100%) as one patient may present with more than one manifestation. SABA: short-acting beta agonist; ICS: inhaled corticosteroids; LABA: long-acting beta-agonist

Characteristics*		n (%)
Age in years; mean±SD (range)		6.1±3.0 (1-14)
Age in categories	<5 years	401 (37.2)
	5-12 years	676 (62.8)
Sex	Male	644 (59.8)
	Female	433 (40.2)
Nationality	Saudi	1009 (93.7)
	Non-Saudi	68 (6.3)
Asthma manifestation**	Shortness of breath	632 (58.7)
	Cough	650 (60.4)
	Chest tightness	459 (42.6)
	wheezing	528 (49.0)
Number of controller medications for asthma	SABA	797 (64.7)
	SABA-ICS	275 (25.5)
	SABA-LABA	57 (5.3)
	SABA-LABA ICS	48 (4.5)
Relief after SABA use	Yes	850 (78.9)
	No	227 (21.1)
Use of systematic corticosteroid	Yes	261 (24.2)
	No	816 (75.8)
Associated co-morbidities	Yes	45 (4.1%)
	No	1032 (95.9)
Family history of asthma/allergy	Yes	748 (69.4)
	No	329 (30.6)
Flu vaccination	Yes	515 (47.8)
	No	562 (52.2)

Table 2 presents patients requiring ED visits/month before and after health education. There have been statistically significant differences regarding patients requiring ED visits/month for all studied patients and among patients <five years of age and those aged 5-12 years. However, the average number of emergency department (ED) visits per patient a month showed no statistically significant difference for all studied groups, although it was reduced in the post-education groups.

**Table 2:** Patients requiring ED visits/month before and after health education program. *Data are presented as n (%) and mean±SD. **P-value is statistically significant.

Outcome*		Pre-education	Post-education	p-Value
All patients (n=804)	Patients requiring ER visits/month	287 (35.7)	216 (22.9)	0.001**
	Average number of ED visits/patient/month	0.15±0.56	0.12±0.40	0.15
Patients <5 years (n=292)	Patients requiring ED visits/month	107 (36.7)	82 (28.1)	0.004 **
	Average number of ED visits/patient/month	0.18±0.43	0.10±0.53	0.32
Patients 5-12 years (n=512)	Patients requiring ED visits/month	180 (35.1)	134 (26.2)	0.001**
	Average number of ED visits/patient/month	0.14±0.34	0.15±0.68	0.86

Table 3 shows the patients requiring hospitalization/years before and after health education. There have been statistically significant differences regarding patients requiring hospitalization/years for all studied patients and among patients <five years and those aged 5-12 years. Although it was lower among the post-education groups, the average number of hospitalization/years showed no statistically significant difference.

**Table 3:** Patients requiring hospitalization/years before and after health education program. *Data are presented as n (%) and mean±SD. **P-value is statistically significant.

Outcome*		Pre-education	Post-education	p-Value
All patients (n=804)	Patients requiring hospitalization/year	198 (24.6)	106 (13.2)	0.01**
	Average number of hospitalizations/patient/year	0.55±1.39	0.19±0.62	0.34
Patients <5 years (n=292)	Patients requiring hospitalization/year	78 (26.7)	41 (14.0)	<0.0001**
	Average number of hospitalizations/patient/year	0.58±1.02	0.21±0.75	0.09
Patients 5-12 years (n=512)	Patients requiring hospitalization/year	120 (23.4)	65 (12.7)	0.003**
	Average number of hospitalizations/patient/year	0.53±0.9	0.19±0.58	0.09

Table 4 displays the patients requiring pediatric intensive care unit (PICU) admission/years before and after health education. There have been statistically significant differences regarding patients requiring pediatric intensive care unit (PICU) admission/years for all studied patients and among patients <five years of age and those aged 5-12 years. Also, average number of PICU/patient/year was markedly reduced among the post-education groups with statistically significant differences.

**Table 4:** Patients requiring PICU admission/years before and after health education program. *Data are presented as n (%) and mean±SD. **P-value is statistically significant. PICU: pediatrics intensive care unit

Outcome*		Pre-education	Post-education	p-Value
All patients (n=804)	Patients requiring PICU/year	40 (4.9)	12 (1.5)	0.01**
	Average number of PICU/patient/year	0.10±0.14	0.01±0.09	0.04**
Patients <5 years (n=292)	Patients requiring PICU/year	18 (6.1)	3 (1.1)	<0.0001**
	Average number of PICU/patient/year	0.11±0.19	0.02±0.10	0.001**
Patients 5-12 years (n=512)	Patients requiring PICU/year	22 (4.3)	9 (1.8)	0.03**
	Average number of PICU/patient/year	0.10±0.12	0.002±0.11	0.001**

Table 5 presents the patients with controlled asthma test (ACT) and Test for Respiratory and Asthma Control in Kids (TRACK) before and after health education. The average ACT and TRACK scores were significantly higher in the post-education groups, and the high percentage of asthma control was higher among post-education groups with significant differences.

**Table 5:** Asthma control before and after health education program. *Data are presented as n (%) and mean±SD. **P-value is statistically significant. ***Total scoring 0-100. ****Total scoring 0-25. ACT: Asthma Control Test; TRACK: Test for Respiratory and Asthma Control in Kids

Outcome*		Pre-education	Post-education	p-Value
Patients <5 years (n=292)	Patients with controlled asthma	18 (6.1)	211 (72.3)	<0.0001**
	Mean TRACK score***	0.11±0.19	90.5±14.6	0.001**
Patients 5-12 years (n=512)	Patients with controlled asthma	216 (31.9)	369 (54.6)	0.008**
	Mean C-ACT score****	14.8±2.1	23.2±4.9	0.004**

Table 6 displays asthma control level and clinical outcome variables after education by flu vaccination status. Among the patients <five years of age, there was only a significant difference between vaccinated and non-vaccinated groups with regard to hospitalization/year where only 7.6% of vaccinated children were admitted to hospital during the period of follow-up compared with 17.6% of non-vaccinated children. Emergency department (ED) visits and pediatric intensive care unit (PICU) admission rates were lower and the average TRACK score was higher among these children. Hospitalization and PICU admission rates were also significantly reduced among vaccinated groups of children aged 5-14 years. Vaccinated children of this age group also showed a higher rate of asthma control and average Childhood Asthma Control Test (C-ACT) score, though not significant.

**Table 6:** Asthma control level and clinical outcomes among asthmatic children after health education by their vaccination status. *P-value is statistically significant. TRACK: Test for Respiratory and Asthma Control in Kids; C-ACT: Childhood Asthma Control Test; PICU: pediatric intensive care unit

Outcome			Flu vaccine		p-Value
			Yes (n=105)	No (n=187)	
Patients <5 years (n=292)	Patients with controlled asthma	15 (14.3)		34 (18.2)	0.39
	Mean TRACK score	91.2±14.9		90.1±14.5	0.52
	ED visits/month	27 (25.7)		55 (29.4)	0.50
	Hospitalization/year	8 (7.6)		33 (17.6)	0.01*
	PICU/year	1 (1.0)		2 (1.2)	0.90
	Vaccination status	Yes (n=239)		No (n=273)	-
Patients 5-12 years (n=512)	Patients with controlled asthma	231 (96.7)		259 (94.9)	0.32
	Mean C-ACT score	23.4±2.8		23.3±3.3	0.79
	ED visits/month	58 (24.3)		76 (27.6)	0.42
	Hospitalization/year	28 (11.7)		47 (17.5)	0.04*
	PICU/year	3 (30.0)		6 (70.0)	0.02*

## Discussion

This cross-sectional intervention design study has analyzed data from 804 asthmatic children in the Madinah region, Saudi Arabia to assess the impact of asthma education programs and influenza vaccination on asthma control levels and clinical outcomes among them. The results of the present study found that the asthmatic patients requiring ED visits/month before and after health education were significantly reduced among post-education groups of all studied patients as well as among patients less than five years and those aged 5-12 years. In their cross-sectional study on 297 asthmatic children patients in two healthcare facilities in Riyadh, Saudi Arabia, Al-Muhsen et al. found that about half of the studied patients (48.2%) visited the ED because of poor education about asthma and/or medication use [15]. The authors concluded that patients educated about asthma were less likely to stop corticosteroid therapy and to visit ED. Another recent cross-sectional study on 250 asthmatic children aged 1-17 years in Jazan province, Saudi Arabia, reported that poor education about asthma and medication use were associated with frequent and unnecessary visits to emergency department and inefficient application of management action plan [16].

Frequent visiting of mild asthmatic patients to the ED, particularly by non-educated patients will maintain the problem of overcrowding and poor utilization of health resources [17]. Criticism of such unnecessary visits is further supported by observations showing that providing regular primary care follow-up for asthmatic children has helped in reducing asthma relapses and decreased ED visits [17,18].

Watson et al. have examined changes in the number of visits to the emergency department during the year after health education intervention and found great reduction in the number of visits compared with that done in the year prior to the intervention [18]. In another study, Cano-Garcinuño et al. showed that the benefits are apparent when education was aimed at children and their parents and no additional benefit was obtained if the intervention was also aimed at their caregivers [19]. These results indicate the importance of providing a complete asthma education plan for both patient and family members and their role in reducing ED visits.

Asthmatic patients requiring hospitalization and PICU admission/years before and after health education program in the present study have shown statistically significant differences where hospitalization and PICU admission have markedly reduced among the post-education groups. A recent quasi-experimental design was conducted in the Ha'il region of Saudi Arabia on 228 asthmatic patients (130 cases and 98 controls) to assess the impact of a school-based, nurse-delivered asthma health education program on asthmatic children's knowledge, quality of life, school absenteeism, and hospitalization showed a significant increase in students' knowledge and school attendance, and decrease in the rate of hospitalization among the studied children [20]. Also, many studies have attributed the hospitalization of asthmatic children patients and their admission to a tertiary hospital to the associated lack of knowledge related to asthma disease and poor asthma control [21]. The major goal of asthma management is to achieve control of the disease and to improve the quality of life of affected children and their families, avoid school absenteeism, increase productivity, and prevent the need for emergency care and hospitalizations [22]. In a multicentre study including 29 tertiary hospitals in China to assess the status of asthma control and severity of asthma in children and to identify impact factors, Zhao et al. reported the association of hospitalization and PICU admission with the parents' knowledge, attitude, and practice towards asthma disease and concluded this factor as a predictor of asthma control level [23]. A randomized control intervention study at Michigan State University, United States on 901 pediatric patients with asthma (458 control and 443 intervention group) has also compared the number of emergency attendance, clinic visits, urgent care visit, hospitalizations, and spirometry before and after education provided by certified asthma educator and reported a decrease in total clinic visits, the need for steroid bursts, hospitalization, and urgent care need after the intervention [24].

In the present study, asthma control level was assessed by C-ACT for children aged 5-12 years, and TRACK for children less than five years old. The study results found that the average ACT and TRACK scores were significantly higher in the post-education groups, and the asthma control level was significantly increased among these children. Consistent with these findings, a recent cross-sectional study was conducted on 278 asthmatic children aged 1-12 years attending the primary health care centers in Madinah, Saudi Arabia, in 2018, to evaluate asthma control levels among them and to investigate the association between asthma control and the knowledge of caregivers. The authors found that increasing the knowledge scores of the caregivers was significantly associated with increased asthma control level scores [25].

Comparing the studied outcome variables by infulnza vaccination status of children, the study findings showed no significant difference with regard to asthma control level and their average score. However, the rate of hospitalization and PICU admission were significantly reduced, particularly among children aged 5-14 years. In a cross-sectional study of 190 parents who presented with asthmatic children at King Fahad Medical City (KFMC) in Riyadh, Saudi Arabia, about 76% of parents with vaccinated children agreed that the flu vaccine could safeguard their children's asthma complications and doctors' opinion about flu vaccination was significantly associated with the parents' decision [26]. In the present study, the rate of hospitalization was significantly lower among vaccinated children. It was 7.6% in the vaccination group of children <five years and 11.7% in children aged 5-14 years. Among the unvaccinated groups, however, the rate of hospitalization was 17.6% and 17.5%, respectively. A similar finding was reported in a cross-sectional comparative study on 368 asthmatic children, of whom 132 (36.5%) received seasonal influenza vaccine where the rate of hospitalization among vaccinated children was significantly lower compared with unvaccinated children [27]. Several studies have investigated the factors contributing to asthma complications among children; in addition to poor air quality and environmental factors, viral infections are the most common triggering factor that leads to asthma exacerbation and complications that required hospitalization and critical care need in children aged 4-10 years [28]. The Ministry of Health in Saudi Arabia has previously designed a management plan to control asthma-associated complications. The program focuses on raising the awareness of the general population through educating and encouraging the parents of asthmatic children to inoculate them with the seasonal influenza vaccine [29].

The strength of this study includes a relatively large sample size, despite the coronavirus disease 2019 (COVID-19) pandemic overlapping with the study period, compared with similar international and local studies. To the best of available knowledge, this study is considered to be the first in Madinah city to assess the impact of asthma education program and flu vaccination on asthma control levels among asthmatic children patients. Moreover, the current study examined not only the impact of education program and flu vaccination on asthma control levels but also the impact on the related clinical outcomes, such as ED visits, hospitalization, and PICU admission.

This study also has a number of limitations. Self-selection bias may have been a limiting factor in this study because poor asthma control is likely to be available while mild and controlled asthma cases may refuse to participate and/or be contacted during such unprecedented period. Also, the excluded asthmatic children was 33.9% and this may be a potential risk for selection bias. However, the socio-demographic and clinical data of these excluded children were comparable with the analyzed asthmatic children (data not shown). Because of the cross-sectional nature of this study, a causal association cannot be confirmed and longitudinal and experimental studies are needed.

## Conclusions

The implementation of a pediatric asthma education program for children and their parents as well as flu vaccination has decreased the ED visits, the need for hospitalization, critical care, and PICU admission with better asthma control scoring after the intervention. The study recommends designing more longitudinal and experimental studies on this issue and increasing public awareness by improving their knowledge about asthma disease and encouraging parents to inoculate their children with flu vaccination which, in turn, helps to decrease asthma exacerbation and asthma-related complications among these children.
